# Editorial: Psychedelic humanities

**DOI:** 10.3389/fpsyg.2024.1474064

**Published:** 2024-08-21

**Authors:** Tehseen Noorani, Oliver Davis, Alex Dymock, Nicolas Langlitz, Anne K. Schlag, Erika Dyck

**Affiliations:** ^1^School of Pharmacy, The University of Auckland, Auckland, New Zealand; ^2^School of Modern Languages and Cultures, University of Warwick, Coventry, United Kingdom; ^3^Law Department, Goldsmiths University of London, London, United Kingdom; ^4^Department of Anthropology, The New School for Social Research, New York, NY, United States; ^5^Division of Psychiatry, Department of Brain Sciences, Faculty of Medicine, Imperial College London, London, United Kingdom; ^6^Department of History, University of Saskatchewan, Saskatoon, SK, Canada

**Keywords:** psychedelic experience, set and setting, context, culture, anthropotechnics, humanities, social science and humanities

## Introduction

Psychedelic knowledge production over the past two decades has predominantly revolved around psychedelic science. However, in recent years an increasing number of humanities scholars and social researchers have begun studying psychedelic drugs and the cultural shifts accompanying the “psychedelic renaissance.” We decided to organize this Research Topic to represent psychedelic humanities research, but also to explore it, to try to get a sense of what kind of scholarship is being produced. We released our call for papers in 2022, at a time when the initial hype surrounding the medicalization of psychedelics came to be countered by an “anti-hype” that highlighted the pharmacological, psychological, and cultural risks of the drugs' emerging uses (Noorani and Martell, 2021; Yaden et al., 2022; Langlitz, 2023).

In our call for this Research Topic, we took inspiration from scholars who have sought to expand the concepts of “set and setting” beyond laboratory and clinical contexts (Eisner, 1997; Hartogsohn, 2020). Such wider “cultural settings” can lead people to question the value of different states of consciousness and guide decisions to take psychedelics in the first place. Questions of what values and meanings people attribute to their experiences, and the interplay between experience and context, are at the heart of humanities scholarship. Moreover, the humanities often differ from the sciences in that they habitually make thematic the conceptual and normative frameworks in which they operate. Indeed, this may go some way to explaining the allure for humanities scholars of psychedelic experiences, with their qualities of self-referentiality, phenomenal richness, and polyvocality. The humanities tend to avoid rigid definitions or outcomes in favor of embracing complexity, even when it may undermine consensus or appear contradictory.

Through this Research Topic, we sought to explore what psychedelics can teach us about themselves, and ourselves, including aspirational experiences alongside cautionary tales about the potential pitfalls of a psychedelic renaissance and what it might come to represent in this era of human history. We invited scholars working in the humanities and social sciences to contribute to ethical, sociological, gendered, historical, anthropological, philosophical, and policy perspectives on the use of psychedelics. While we welcomed studies that explore intersections of medicine and culture, we especially encouraged submissions from scholars examining psychedelics in non-medical settings, including ceremonial spaces, recreational settings, or spaces where consumers seek psychedelic experiences in order to expand notions of human flourishing and creativity.

In what follows, we survey how the articles of the Research Topic illustrate diverse topics and approaches within the psychedelic humanities, point to some gaps in the Research Topic, and finally consider the possibility of a more programmatic description of the field. With respect to a programmatic for the humanities as inherited from the 19th century, philosopher Sloterdijk (2009) has argued for the centrality of books as “anthropotechnical” devices for reflexive use by humans in exploring and augmenting our common humanity. We wonder whether the psychedelic humanities today might approach psychedelics as analogously educative, offering a set of anthropological devices through which psychedelic experiences can come to reveal ourselves to ourselves, and to offer up new clay for our self-fashioning. While the “humanizing” mission of the humanities was and often still is articulated in restrictive and non-inclusive terms—which are rightly criticized in reflections on the limitation of the category of the human (Wynter, 2003; Cordova)—the promise of individual and collective betterment through anthropotechnical devices such as books and psychoactive substances remains.

## Surveying the articles

The 20 published articles are a good sample of the coalescing field of the psychedelic humanities: eclectic, effervescent and clearly flourishing! We received submissions across a range of disciplinary approaches, from history to cultural and literary studies, from philosophy to black studies, and from bioethics to psychology. Perhaps unsurprisingly, the majority of the articles occupy themselves with the dominant mode of knowledge production in psychedelic scholarship: psychedelic science. This includes synthesizing scientific claims (e.g. Greń et al.), using extra-scientific concepts and arguments to reinterprete scientific data (Kähönen; Devenot and Erving), and using scientific findings as a jumping-off point for asking questions about meaning, the nature of consciousness, or ethics (Jylkkä; Spriggs et al.; Greń et al.). Philosophical contributions offer meta-frameworks and engage in “conceptual hygiene” work (Meling and Scheidegger; Cea; Langlitz), while critical analyses seek to dwell in asking better questions rather than rushing to provide poorly formulated answers (Cordova). In some of these engagements with psychedelic science, we note how authors might hedge bets on the state of the science. For instance, in taking a received trope from psychedelic science and running with it, humanities scholars can hope to articulate particular tensions, antagonisms, and nascent possibilities in psychedelic science, but at the risk of tethering their own relevance to the variable shelf lives of the scientific claims they rest atop.

Beyond psychedelic science, articles also look ahead to worlds where psychedelic-assisted therapy (PAT) is provided at scale, considering what PAT delivery and wider healthcare provision could and should look like, as well as the processes that might best enable us to get there (Spriggs et al.; Greń et al.; Sjöstedt-Hughes; Jacobs). Reviews of scientific research programs are also used to pave the way to remembering or reanimating neglected scientific trajectories (Davis), while focusing in on specific histories of science garners fresh insights into the contemporary cultural politics of psychedelia (Dubus et al.; Jones).

Moving into the macrosociological and macropolitical, we find a discursive analysis of the multidisciplinary problems and concerns that have shaped the framing of cognitive liberty (González Romero), and studies of spiritual technologies—both the persistence of “evolutionary spirituality” within Western psychedelic culture that has often enabled dogmatism and “spiritual eugenics” (Evans), and the liberatory possibilities and pitfalls of the nexus of psychedelic and digital (or “cyberdelic”) technologies (Hartogsohn). Finally, there are studies on the coloniality of psychedelic music therapy (Ratkovic et al.), and on the ongoing impact of neoliberalism on psychedelic experiences (Sanchez Petrement), including the possibility of psychedelic-assisted group therapy as a solution to the problem of neo(liberal)-nihilism (Plesa and Petranker).

An eclectic mix of evidence reveals diverse disciplinary trainings, ranging from the extensive use of footnotes in ways that refuse linearity and unifocality in the text (Davis), to the use of tables, figures, and charts as is common within psychedelic science. Indeed, a selection of articles are penned by psychedelic scientists moonlighting as psychedelic humanities scholars (e.g. Meling and Scheidegger; Spriggs et al.). Given also that many of the articles are in direct conversation with psychedelic science, this raises the possibility of the psychedelic humanities as a genre that does not only have to be served by humanities scholars but could also be a space for scientists to discuss what cannot be said in the constraints of scientific publications. Indeed, might the contributions of psychedelic scientists to the psychedelic humanities be greater than, for example, the contributions of environmental scientists to the environmental humanities, or that of medical researchers to the medical humanities? If there is a markedly greater enthusiasm on the part of psychedelic scientists to weigh in on questions more proper to the humanities, we wonder if this tells us something about the agency of psychedelic substances and experiences in convening interdisciplinary inquiry around them.

## What's missing?

There were notable gaps in the Research Topic. Discussions of gender and sexuality, indigenous knowledges, analyses and practices, and race and colonialism, were less prominent than we had hoped (see Cordova and Ratkovic et al. for exceptions). This perhaps reflects the venue and the costs, particularly for early career researchers, associated with the Frontiers publication model. Regardless, we look forward to future collections on the psychedelic humanities taking up these themes in much more central ways. We also saw very little consideration of psychedelics and embodiment, a relative absence that marks the possibility of a resistance to the overly-mentalistic/neurocomputational orientation of the biomedical research (see Davis, Unpublished manuscript, ch.3). In terms of the emergence of the psychedelic healthcare industry, we were struck that there was little explicit call for a slowing down—if not outright abandonment—of psychedelic medicalisation processes, as has been articulated in other collections (e.g. Hauskeller and Schwarz, 2023).

One of the questions raised in our call as a provocation for authors was, “How does qualitative evidence enhance or detract from our understanding of psychedelic studies?” No articles addressed this directly, other than through close textual analysis of individual psychedelic experiences (Davis; Devenot and Erving). The standard fare of microsociological work on “drug events” as heavily shaped by Bruno Latour and the field of science and technology studies was missing from the Research Topic. On the one hand, this underscores the need to build bridges between the emerging psychedelic humanities and the critical drug studies literature (e.g. Fraser et al., 2014). On the other hand, we wonder what this tells us about access to psychedelic lab sites, whose proliferation is surely the most significant sociological development of the past several years. Some of us have first-hand experience of securing prolonged periods of time in psychedelic labs and patient group networks and imagine that these opportunities may be becoming more difficult to obtain and manage, as the chances of reputational damage from critical researchers to psychedelic corporate research ventures is considered by the latter to be too risky. Yet reverting to knowledge production that does not require this kind of empirical work, particularly without friendships and relations of trust with psychedelic scientists to fall back on, may stymie future scholarship from growing beyond the impasses and polemical tensions found in the extant peer-reviewed literature.

## Toward a programmatic description of the psychedelic humanities

We edited this Research Topic at a time when the overzealous celebration of psychedelic medicalisation had begun to lead to an equally overzealous “anti-hype” reaction. While critique—and even criticism—offer valuable nuance in the face of unbridled optimism, there is a risk of this giving way to an entrepreneurship of psychedelic negativity dominating the field of the psychedelic humanities. With this in mind, we also sought to parse the Research Topic for positive visions of the role of the psychedelic humanities beyond, for example, keeping the hubristic excesses of the psychedelic sciences in check.

A curatorial role could be in finding fruitful alliances across the research of different humanities and social science disciplines. Articles that focus on risks in PAT delivery might appeal to bioethicists who are concerned with risk-management, while specific suggestions for testable interventions might resonate better with psychedelic research labs than with high-level critiques of neoliberalism. In turn these political critiques might inform—and be informed by—policy discussions around the scaling of psychedelic therapies. These alliances may reflect matters of scale, with some disciplines exploring the real-time implications of applied psychedelics, while others take a wider perspective in an effort to refocus our attention on risks and benefits over a long durée.

Returning to the Sloterdijkian framework of anthropotechnics, Davis wonders if psychedelics might be best construed as “tools, techniques, or technologies for the modification of the human,” alongside books and other cultural objects, to “learn from the experience, refine, and repeat in an elevating cycle of practicing to develop performance and yield.” Several other articles in the Research Topic frame psychedelics as tools or technologies in ways that offer the potential of (self-)education (cf. Foucault, 1988; see for example, Devenot and Erving; Hartogsohn; Kähönen; Evans; González Romero). As a field for the exploration of these potentials, could the psychedelic humanities be a place for the psycho-formative education of the self through our relationships with psychedelics? This orientation to psychedelics as pedagogical tools might offer fresh resources for approaching the challenges posed by the ethics of informed consent in relation to “unknowable” experiences.

Langlitz calls for the psychedelic humanities to be a place where scholars might “refrain from offering normative orientation and instead increase the complexity of the observed phenomena by opening other possible perspectives, leaving it to their readers to reduce the resulting complexity in novel ways.” As such, Langlitz argues, “The goal of the psychedelic humanities is to sharpen the sense of possibility and expand the imagination of the psychedelic renaissance.” We recall here Markus' (1987, p. 34–35) useful description of the humanities as existing in a “polemic-dissensive manner” (Langlitz, 2019), in contrast with the tendency of the sciences to be consensus-oriented. Many of the papers certainly speak to and through various -isms: psychedelic humanism, naturalism, spiritual evolutionism, mysticism, neoliberalism. Indeed, Sjöstedt-Hughes's article explicitly develops a typology for many contrasting philosophical frameworks to mysticism. With this variation in mind, the psychedelic humanities could be a zone for the development of many frameworks, each charged with competing values, norms, metaphysical assumptions and so on. Scientific naturalism would be parochialized as one amongst many, and the psychedelic humanities would provide a space for thinking about how these divergent frameworks relate to each other. This is consistent with the call at the end of Langlitz that the psychedelic humanities today, at a time of polarization, could strive for mind-loosening and noncommitment.

But another term for “mind-loosening” is “psycholytic,” suggestive of a lower-dose version of what psychedelic humanities could be. At the risk of inviting greater political agonism into Langlitz's formulation of psychedelic humanities-as-mind-loosening, some of our articles appear to represent a more high-dose “psychedelic” humanities, in line with a model of psychedelic drug action that strengthens high-level beliefs (Safron, 2020; as cited in Meling and Scheidegger) and commitments. We could also go the other way, to consider a “microdosing” humanities as representing more invisible, infrastructural forms of augmentation that enable new intellectual articulations to take place: backstage relationship-building and cross-disciplinary, interdisciplinary, and transdisciplinary alliances, partnerships and pacts. In yet another spiral engagement with psychedelic science, then, emerging doxa around dose-related interventions (see Garcia-Romeu and Richards, 2018, in relation to psycholytic vs. psychedelic interventions), may enable new recursions in the dynamic between the psychedelic sciences and the scholarship of the psychedelic humanities. Indeed, perhaps this might comprise the next iteration of a Research Topic of this sort.

## Conclusion

These are exciting times for the psychedelic humanities. Psychedelic science is becoming increasingly heterogeneous—through ever-more-sophisticated basic scientific research, the increasingly complex array of tactics and strategies by which industrial psychedelic science is producing both knowledge and ignorance (Proctor and Schiebinger, 2008), and more creative local psychedelic trial designs that strike the balance between controlled experimentation and real-world evidencing afresh as psychedelic medicine is normalized and barriers to entry for research are diminished.^1^ The psychedelic humanities will no doubt engage with all of this, but also find its own pathways, in search of new modes of flourishing in the intimacies of our relationships with and through psychedelic substances. This Research Topic has reminded us that the psychedelic movement (such that it is) needs both the sciences and the humanities to effectively evaluate different kinds of evidence, to (re)imagine diverse psychedelic use practices, and to confront the inherent complexities and contradictions that emerge when working across historical, cultural, political and scientific registers.

## Footnotes

^1^For example, see https://tuwairua.org.
